# Trading off accuracy and explainability in AI decision-making: findings from 2 citizens’ juries

**DOI:** 10.1093/jamia/ocab127

**Published:** 2021-08-01

**Authors:** Sabine N van der Veer, Lisa Riste, Sudeh Cheraghi-Sohi, Denham L Phipps, Mary P Tully, Kyle Bozentko, Sarah Atwood, Alex Hubbard, Carl Wiper, Malcolm Oswald, Niels Peek

**Affiliations:** 1 Centre for Health Informatics, Division of Informatics, Imaging and Data Science, Manchester Academic Health Science Centre, The University of Manchester, Manchester, UK; 2 NIHR Greater Manchester Patient Safety Translational Research Centre, School of Health Sciences, Manchester Academic Health Science Centre, The University of Manchester, Manchester, UK; 3 Division of Pharmacy and Optometry, School of Health Sciences, The University of Manchester, Manchester, UK; 4 Division of Population Health, Health Services Research & Primary Care, School of Health Sciences, The University of Manchester, Manchester, UK; 5 Jefferson Center, Saint Paul, Minnesota, USA; 6 Information Commissioner’s Office, Wilmslow, UK; 7 School of Law, Faculty of Humanities, The University of Manchester, Manchester, UK; 8 Citizens’ Juries CIC, Manchester, UK

**Keywords:** artificial intelligence, choice behavior/ethics, citizens’ jury, public opinion, qualitative research

## Abstract

**Objective:**

To investigate how the general public trades off explainability versus accuracy of artificial intelligence (AI) systems and whether this differs between healthcare and non-healthcare scenarios.

**Materials and Methods:**

Citizens’ juries are a form of deliberative democracy eliciting informed judgment from a representative sample of the general public around policy questions. We organized two 5-day citizens’ juries in the UK with 18 jurors each. Jurors considered 3 AI systems with different levels of accuracy and explainability in 2 healthcare and 2 non-healthcare scenarios. Per scenario, jurors voted for their preferred system; votes were analyzed descriptively. Qualitative data on considerations behind their preferences included transcribed audio-recordings of plenary sessions, observational field notes, outputs from small group work and free-text comments accompanying jurors’ votes; qualitative data were analyzed thematically by scenario, per and across AI systems.

**Results:**

In healthcare scenarios, jurors favored accuracy over explainability, whereas in non-healthcare contexts they either valued explainability equally to, or more than, accuracy. Jurors’ considerations in favor of accuracy regarded the impact of decisions on individuals and society, and the potential to increase efficiency of services. Reasons for emphasizing explainability included increased opportunities for individuals and society to learn and improve future prospects and enhanced ability for humans to identify and resolve system biases.

**Conclusion:**

Citizens may value explainability of AI systems in healthcare less than in non-healthcare domains and less than often assumed by professionals, especially when weighed against system accuracy. The public should therefore be actively consulted when developing policy on AI explainability.

## INTRODUCTION

The use of artificial Intelligence (AI) is on the rise in healthcare as well as other domains, such as agriculture and finance.^1^ Modern AI systems typically rely on machine learning, a technology that automatically constructs computer systems through real-world experiences captured in large volumes of data.^2^ Deep learning is at the forefront of this development: a 2019 meta-analysis of 25 studies of AI diagnostic systems in medical imaging and histopathology concluded that the diagnostic performance of deep learning models was equivalent to that of healthcare professionals.^3^ This may explain why some people anticipate that AI systems will create new roles for doctors as information specialists, or even replace entire medical disciplines.^4^ However, due to their complex internal structure, deep learning models are generally considered “black boxes” that do not providing information about how exactly they arrive at their decisions.

Some feel uncomfortable with black box AI. They argue that for sensitive decision-making tasks affecting human well-being and health, it is not acceptable to use AI systems that are not transparent and whose reasoning cannot be understood by those affected by the decisions.^5^ This not only undermines trust but also reduces possibilities for verifying that the reasoning methods are sound and robust and that the resulting decisions are safe and fair.^6^

However, more explainable AI systems may be less accurate and vice versa.^7^ Viewed as such, AI explainability becomes a double-edged sword: while more explainability enhances the opportunity to verify and contest a decision, it would also increase the probability of error. The person affected by that decision may therefore not necessarily consider increased explainability a good thing. To date, no studies have explored how people would make that trade-off between accuracy and explainability of AI systems used to make decisions about them.

Currently, there is no clear guidance on whether AI-based decisions should be accompanied by an explanation. There are position statements emphasizing the importance of explainability (or transparency) of AI decision-making,^8^ and the “right to explanation” is also a subject of ongoing legal debate. Under the United States Equal Credit Opportunity Act, creditors are required to provide specific reasons when denying someone a loan.^9^ The European Union General Data Protection Regulation, Recital 71, states that when people are subject to decisions based solely on automated processing, they have a right to obtain an explanation of the decision reached.^10^ But position statements and recitals are not binding, and the right to an explanation has been removed from the binding articles of the text during the legislative process. This has resulted in a scarcity of policy on if and how developers, users, and regulators should ensure AI explainability.

Therefore, we organized 2 citizens’ juries, a method to explore what members of the public think about a policy problem after they have been well informed. Our juries addressed the question: “Should AI systems give an explanation, even if that results in less accurate decisions?” The juries aimed to inform guidance on explaining processes, services, and decisions delivered or assisted by AI to the individuals affected by them. This article reports on the mixed-methods study conducted alongside the juries, which investigated how the public makes the trade-off between accuracy and explainability of AI, their reasoning behind this, and whether this differed between healthcare and other domains.

## MATERIALS AND METHODS

### Citizens’ juries to engage the public in policy making

Developed in the 1970s by the Jefferson Center,^11^ citizens’ juries are a form of deliberative democracy: an egalitarian approach to public policy making that encourages mutual recognition and respect and allows public negotiation for the common good.^12^ In a citizens’ jury, a representative sample of individual citizens (ie, jurors)—from different backgrounds and without having special prior knowledge or expertise—come together for several days, hear expert evidence, deliberate together and reach reasoned conclusions about the public policy questions posed to them. This approach assumes that jurors can answer these questions once they are properly informed about available evidence and are encouraged to deliberate in an environment free of “delusion, deception, power, and strategy.”^13^^,^^14^ Citizens’ juries are particularly effective when there is a values trade-off at the heart of the questions they are being asked. As such, they have been used for informing health policy on ethically complex topics, such as genetic testing,^15^ screening services,^16^ and case finding for stigmatized diseases.^17^ Our research group previously organized 3citizens’ juries on health data sharing for research and commercialuse.^18–20^

### General approach

In February 2019, we organized 2 citizens’ juries in the UK, 1 in Manchester and 1 in Coventry. Each lasted 5 days and involved 18 jurors, without overlap in participants between the juries. This sample size was convenient for smaller and larger group work (eg, 6 groups of 3 and 3 groups of 6) and aimed to enable all jurors to actively take part in the discussions. Both juries followed an identical process, allowing us to systematically compare results between the 2 juries to strengthen the robustness of our findings. The running of the jury was contracted to Citizens’ Juries c.i.c.,^21^ a social enterprise that managed the project, designed and facilitated the jury process in partnership with the Jefferson Center, and recruited jurors, expert witnesses, and an independent oversight panel. The 3 oversight panel members were chosen for their topic knowledge and lack of conflict of interest in a particular jury outcome. They reviewed all jury materials (scenarios, witness briefs, presentations, questionnaires, planned activities) to ensure scientific rigor and identify bias in the jury design toward either accuracy or explainability. To further minimize risk of bias in our findings, we strictly separated roles within the study team: those who were involved in the design and conduct of the citizens’ juries (NP, KB, SA, MT, MO) were not involved in the qualitative data collection, analysis, and interpretation (LR, SCS, DP, SvdV).

The study was approved by The University of Manchester Research Ethics Committee (Ref: 2019-6023-9065).

### Recruitment

We invited citizens to apply as a juror via adverts on a job website (www.indeed.co.uk) for both cities as well as a volunteering website (https://www.vacoventry.org.uk) in Coventry. For each jury, we created a shortlist of 18 applicants through stratified sampling, ensuring that the shortlist matched the latest UK Census Data for England^22^ in terms of age, gender, ethnicity, educational attainment, and employment status. We also aimed to match their prior views on the topic to the responses of 2074 adults participating in a survey commissioned by the Royal Society for the encouragement of Arts, Manufactures, and Commerce^23^ to the question “*How comfortable, if at all, are you with the following idea? As the accuracy and consistency of automated systems improve over time, more decisions can be fully automated without human intervention required.*”

Shortlisted applicants were interviewed by telephone to ensure that they: (i) did not have substantial prior knowledge or expertise on the jury topic, or a special or conflicting interest; (ii) understood what was being asked of them; and (iii) were available in the jury week. Participants received £500 for their 5 days “jury service” plus £25 cash expenses.

### Jury program

Based on the jury questions, Citizens’ Juries c.i.c. determined jurors’ information needs and developed the program accordingly. This included a brief for expert witnesses, which guided witness selection. The witness brief and slides are publicly available, alongside all other jury materials and documentation.^24^ Box 1 shows the 5-day jury program, which was a mix of plenary sessions and small group work (see Supplementary Part 1 for detailed jury program). The plenary sessions primarily consisted of presentations by expert witnesses followed by questions from the jurors and of whole group feedback of the small group work. Some expert witnesses argued for a specific position (ie, partial witnesses), while others gave a neutral, balanced account of the topic (ie, impartial witnesses). Witnesses either attended the jury in person or appeared via video link. In both juries, jurors were given the same materials, heard the same presentations, and could ask questions of all expert witnesses. Both juries were led by the same 2 independent facilitators (KB, SA) from the Jefferson Center.

On days 3 and 4, we presented the jurors with 4 scenarios involving applications of AI, 2 of which were related to healthcare (stroke and kidney transplant scenarios) and 2 that were not (recruitment and criminal justice scenarios; see Box 2).

The scenarios were developed by Citizens’ Juries c.i.c. with input from the organizations who commissioned the juries (see Funding statement). The iterative development process was guided by the role of the scenarios within the program, which was to enable jurors to explore the questions and trade-offs of interest. Scenarios reflected realistic settings where AI could be deployed. In each scenario, we described 3 types of automated decision-making systems (systems A, B, and C) with different levels of explainability and accuracy (Box 3). We chose the accuracy levels of these systems so that they offered clear and consistent choices across the scenarios, rather than necessarily reflecting real-world AI performance levels (Supplementary Part 2 presents the complete scenarios). Per scenario, expert witnesses explained the scenario and the systems, after which jurors discussed the (dis)advantages of the systems in small groups and noted these on flip charts. All jurors then indicated on the flipcharts which (dis)advantages they found most important for each system, and anonymously selected their preferred system through electronic voting, including up to 3 reasons in free text to support their preference.

### Data collection and analysis

We collected quantitative and qualitative data for analysis:

- Transcriptions: as part of documenting the jury process, we audio-recorded and transcribed plenary sessions to capture questions raised by jurors, and arranged them by scenario and topic.
- Electronic voting data: anonymized jurors’ votes by scenario, and up to 3 reasons, recorded as free text, to support their choice.
- Observational field notes: qualitative researchers (LR, DP, SCS) observed the plenary sessions and group discussions, and captured their observations as field notes.
- Output from the group discussions on (dis)advantages of each system per scenario: this included jurors’ statements agreed during the small group work, and for each statement how many jurors considered it the most important factor for (not) preferring a particular system.

To investigate how jurors made the trade-off between accuracy and explainability of AI, and whether this differed between healthcare and non-healthcare scenarios, we descriptively summarized the quantitative voting results across the 2 juries per scenario. To understand the reasoning behind their trade-off, we qualitatively analyzed jurors’ free text voting data, researchers’ field notes, and jurors’ statements on (dis)advantages of the systems. For this, LR imported the data into NVivo (QSR International Pty Ltd Version 12, 2018) for management of initial coding and read the data as part of the familiarization process. LR conducted an initial round of conceptual coding using an inductive open coding approach^26^ and organized codes into themes.^27^ SCS independently coded a random 10% sample of the data. The qualitative team (LR, SCS, DP) discussed the results from this consensus exercise, resolving coding differences by majority decision. LR then recoded the data in a second coding round according to the agreed revised coding scheme. The qualitative team met to discuss grouping codes into overarching themes by scenario, both per and across AI systems. These themes were iteratively verified and refined a final time through discussions between LR and NP (Supplementary Part 3 presents an example of how we went from initial coding to theme generation).

## RESULTS

### Jurors

In total, 451 people applied for the juries (271 in Manchester, 180 in Coventry). Thirty-six were selected as jurors (18 per location), of whom most (89%) were recruited via the job website. Table 1 presents juror characteristics, which were largely comparable to those of the English population.

**Table 1.** Juror characteristics compared to the population of England^22^

Characteristics	Jurors (n = 36)	England
	Value (%)^a^	%
*Age (years)*		
18–29	8 (22)	21
30–44	9 (25)	26
45–59	9 (25)	25
60 or older^b^	11 (31)	28
*Gender*		
Male	18 (50)	49
Female	18 (50)	51
*Ethnicity*		
White	27 (75)	85
Non-white	9 (25)	15
*Educational attainment* ^c^		
0 to 4 O levels/GCSEs	10 (28)	36
5 or more O levels/GCSEs	5 (14)	21
2 or more A levels	9 (25)	16
Graduate degree or higher	12 (33)	27
*Employed or self-employed*	22 (61)	76^d^
*Response to pre-jury screening question on the trade-off between accuracy versus explainability* ^e,f^		
Not at all comfortable	8 (22)	26
Not very comfortable	15 (42)	38
Fairly comfortable	8 (22)	23
Very comfortable	2 (6)	3
Don’t know	3 (8)	9
*Recruitment route*		
Job website	32 (89)	n.a.
Voluntary action website	2 (6)	n.a.
Word of mouth	2 (6)	n.a.

*Abbreviations:* GCSEs, general certificate of secondary education; n.a., not applicable.

a Percentages may not add up to 100% due to rounding.

b People of 75 years and older were the lowest users of the internet in the UK in 2019 (47% compared to 91% overall) [28], which means they might have been underrepresented in this age group.

^c^“O levels” and the examinations that replaced them (GCSEs) are UK academic qualifications in a particular subject, typically taken at age 16. “A levels” are a higher level of academic attainment that give access to university, usually taken at age 18. Students typically take up to 8 GCSEs/O levels, and up to 3 A levels. Many students leave education at aged 16, especially in the 1940s to 1980s. A graduate degree is a university degree.

^d^% of people in the UK aged 16–64 years who were (self-)employed in the period Oct–Dec 2018.

^e^The question was formulated as follows: “*How comfortable, if at all, are you with the following idea? As the accuracy and consistency of automated systems improve over time, more decisions can be fully automated without human intervention required.*”

^f^Percentages for England refer to responses of 2074 adults to this question as part of a survey commissioned by the Royal Society for the encouragement of Arts, Manufactures, and Commerce [23].

### How jurors made the trade-off between accuracy and explainability of AI

Table 2 displays the results of the quantitative analysis of the electronic voting per scenario across the 2 juries. For both healthcare scenarios, a large majority (86% and 92% for the stroke and kidney transplantation scenarios, respectively) voted for system C, implying they favored accuracy over explainability in these contexts. Jurors’ votes for related issues (ie, questions 1 and 2) also reflected this preference, although less starkly for question 1, where, for the kidney transplantation scenario, 36% of jurors found it at least fairly important for an individual to receive an explanation of an automated decision. For the non-healthcare scenarios, the majority of jurors made the trade-off differently: they either weighed accuracy and explainability equally by voting for system B or—particularly in the criminal justice scenario—put more emphasis on explanation. Voting results were comparable between the Manchester and Coventry juries, except for the recruitment scenario; whereas the Manchester jurors distributed their votes equally across the 3 systems, 14/18 of Coventry jurors voted for system B (see Supplementary Part 5 for votes per jury).

**Table 2.** Electronic voting results per scenario across the 2 juries; total N = 36, values are numbers (percentages)

	Healthcare Scenarios		Non-Healthcare Scenarios	
Questions	Stroke	Kidney Transplantation	Recruitment	Criminal Justice
*Question 1: How important is it for an individual to receive an explanation of an automated decision?*				
Very important	4 (11)	1 (3)	8 (22)	17 (47)
Fairly important	6 (17)	12 (33)	16 (44)	9 (25)
Not very important	21 (58)	18 (50)	11 (31)	8 (22)
Not at all important	4 (11)	5 (14)	1 (3)	2 (6)
Don’t know	1 (3)	0 (0)	0 (0)	0 (0)
*Question 2: If system C was chosen [by the NHS], almost no explanation would be provided. How much does this matter?*				
Very much	4 (11)	1 (3)	10 (28)	17 (47)
Quite a lot	5 (14)	3 (8)	12 (33)	9 (25)
Not very much	24 (67)	20 (56)	13 (36)	7 (19)
Not at all	3 (8)	12 (33)	1 (3)	3 (8)
Don’t know	0 (0)	0 (0)	0 (0)	0 (0)
*Question 3: Which automated decision system do you think the NHS should choose/should be chosen?* ^a^				
System A—expert system	0 (0)	1 (3)	7 (19)	15 (42)
System B—random forest	5 (14)	2 (6)	20 (56)	13 (36)
System C—deep learning	31 (86)	33 (92)	9 (25)	8 (22)

a System A, expert system (below human expert-level accuracy, full explanation); System B, random forest system (human expert-level accuracy, partial explanation); System C, deep learning system (beyond human expert-level accuracy, no explanation).

### Jurors’ reasoning when making the trade-off between accuracy and explainability of AI

Three themes emerged from our analysis of the qualitative data. Each consisted of 3 subthemes reflecting factors that jurors considered when voting. We discuss the subthemes in more detail below, with Table 3 providing illustrative quotes from the free-text voting data, researcher field notes, and jurors’ statements on (dis)advantages of systems (see Supplementary Part 6 for more juror statements).

**Table 3.** Quotes and statements to illustrate jurors’ considerations when making the trade-off between accuracy and explainability of AI systems^a^^,^^b^

	Healthcare Scenarios		Non-Healthcare Scenarios	
(Sub)Themes	Stroke	Kidney Transplantation	Recruitment	Criminal Justice
Theme A: ACCURACY OF DECISION MAKING				
1. Impact on the individual	“[System C] will save lives” (M, G) “We have a moral and ethical obligation to improve speed and accuracy rates in diagnosis” (C, V, 11) “The explanation comes secondary to accuracy, as without an accurate diagnosis there would be no basis foundation for treatment/aftercare” (C, V, 10)	“Higher accuracy level so less chance for an unsuccessful transplant” (M, V, 59) “When you win the lottery you just need to know that you won, not which order the balls come up” (C, G) “[System C would] save valuable time for the patients, as they would not potentially be given a kidney that would be rejected and go back in the waitlist” (C, V, 10)	“[System B] has key points/features for detection.” (C, G)	“Accuracy of the decision is paramount, as it may determine the individual’s future” (M, V, 68) “The accuracy level [of system A] is the same as a human, but quicker” (M, V, 59)
2. Impact on society	“It would be able to … possibly free up more time for experts to … do more research” (C, G)	“It is important to get as accurate a match as possible to eliminate waste of a kidney that could have potentially helped another [recipient]” (M, V, 65)	“[System B has] high level of possibility finding correct candidate” (C, V, 14)	“[System C has a] lower risk to [the] public” (C, V, 20) “It is important to identify who will reoffend” (M, V, 53)
3. Increased efficiency	“[System C could] save money and time in the long term” (M, V, 56) “[System C] frees up doctors for other duties” (C, V, 16)	“[System C] could reduce costs for the NHS in respect of rejection and fewer drugs prescribed post-operative[ly]” (C, V, 21)	“[System C] gets through large amounts of CVs in a short space of time” (M, V, 59)	“[System A] would require a lot of work from software engineers and policemen” (C, G)
Theme B: EXPLAINABILITY OF DECISION MAKING				
1. Learning opportunities for the individual	“According to [the evidence], most stroke victims don't usually ask how the stroke was diagnosed” (C, V, 17) “More information would lead to better aftercare [and could support] prevention of repeat of stroke” (C, V, 31) “Patient rights to know how the system computed” (M, V, 67) “Doctors wouldn’t trust themselves when presented with a machine that is almost always right and would sometimes incorrectly follow the machine’s advice especially when the machine offers no explanation at all.” (C, V, 24).	“No transparency to show the criteria used (BMI, blood pressure, lifestyle) factored into the decision” (C, G)	“Less transparent feedback [in system B]—eg, could tell you that you didn’t meet essential criteria but not tell you which part you did not meet to improve future chances of success” (C, G) “Gives me motivation to improve and hone my skills for the future” (C, V, 26)	“It could rehabilitate a person who is in real need of direction” (M, V, 51) “[System A is the] best system to provide transparent reasoning for individuals where they have specific factors to focus on during rehab/prosecution” (C, V, 18)
2. Learning opportunities for society	“[With system C, you] can’t interrogate the system so can’t improve the result” (M, G)	n.a.^c^	“Feedback is essential for improving results for candidates, employers, and the economy” (C, V, 16)	“[System A allows] clear decision-making process providing transparency for offender, victim, and any potential further victim” (C, V, 11) “No learning from data [with system C]” (M, V, 71)
3. Ability to identify/resolve bias	n.a.^c^	“Data going in to this system would be hugely skewed in favour of those privileged with existing wellness … [leading to widening] health inequalities” (C, V, 16)	“[System B] can somehow identify biases in the attributes which can be rectified” (C, V, 18) “The factors assessed by systems B and C may be entrenched in historical bias … whether someone is a high flyer precludes factors that make a great employee, like honesty and attitude” (M, V, 55)	“Accuracy is less important because the data [are] so subjective anyway” (C, V, 19) “The custody sergeant will have experience and understanding of offenders and possibly what causes them to offend” (M, V, 51) “[System C] removes individual bias made by individual officers in each respective case” (C, V, 10)
Theme C: TRUST IN AUTOMATED SYSTEMS				
1. Fairness	“There are not specialist doctors available 24 hours a day to reach a decision” (M V, 59)	“[System C] is the fairest—it shortlists on organ suitability alone” (M, V, 55)	“[System B] is ‘democratic’ in its analysis of past applicants, both successful and unsuccessful, without developing unknowable deep learning iterations” (M, G)	“I don’t think something as abstract as potential to reoffend can be discerned by AI” (M, V, 55)
2. Delivery of the decision	n.a.^c^	“[System C] doesn’t take away the sympathetic and empathetic aspect patients need throughout the whole process, but still assists doctors in decision making” (C, V, 18)	n.a.^c^	“[With system A] there is more interaction with officers. Feelings and remorse will be taken into account and a full explanation will be given for the end result” (M, G) “[System A] offers better dialogue between the authority and the offender with clearly defined parameters” (C, G)
3. Accountability for decisions	“[With system C] who is responsible if the diagnosis is wrong with fatal consequences?” (C, G)	“[An explanation is] only relevant for cases that go wrong and an enquiry is launched” (C, V, 11) “There is no recourse when something goes wrong” (C, V, 16)	n.a.^c^	n.a.^c^

*Abbreviations:* AI, artificial intelligence; BMI, body mass index; n.a., not applicable.

a For each quote/statement, the information in brackets refers to the jury location where it was captured (M=Manchester, C=Coventry), and whether it was raised during small/plenary group discussions (G) or as free text during electronic voting (V). In case of the latter, we also provided the participant ID.

b System A, expert system (below human expert-level accuracy, full explanation); System B, random forest system (human expert-level accuracy, partial explanation); System C, deep learning system (beyond human expert-level accuracy, no explanation).

c Subtheme did not emerge for this scenario.

#### Theme A: Accuracy of decision-making

1. *Impact on the individual about whom the decision is being made*; in the healthcare scenarios, the impact on individuals included issues such as preservation of life after stroke and reducing the risk of kidney rejection. For the kidney transplantation scenario, jurors felt that patients primarily needed to know that they had been matched for a transplant, with the explanation of why they were matched being secondary. Furthermore, they considered AI accuracy intrinsically linked to the speed with which healthcare professionals could act. Impact on the individual and speed of decision-making were also deemed important in the criminal justice scenario, with many jurors being surprised at how long people had to wait until their case went to court. For the recruitment scenario, jurors placed little emphasis on accuracy because they didn’t see there was necessarily 1 “best person for the job.”
2. *Impact on society*; for both healthcare scenarios, jurors felt increased accuracy could reduce waste, for example in terms of freeing up time of experts or fewer rejected kidneys. For the recruitment scenario, jurors thought AI primarily contributed to recruitment companies’ reputation by improving their ability to place the right candidate in the right role—but without this necessarily benefitting job applicants. When considering the impact on society for the criminal justice scenario, jurors linked improved accuracy to a reduced reoffending risk.
3. *Increased efficiency*; in the stroke scenario and for both non-healthcare scenarios, jurors viewed fully automated AI systems as a way to save time and resources. For the transplantation scenario, they considered the time gain less critical, and emphasized the potential to reduce costs for the NHS.

#### Theme B: Explainability of decision-making

1. *Learning opportunities for the individual*; for the stroke scenario, jurors felt there was little need for an explanation of the diagnosis because providing an explanation was not part of current clinical practice. However, with regard to a fully automated AI system, they expressed concerns around doctors’ over-relying on the new technology and thereby losing valuable skills. One juror also mentioned patients’ general rights to be informed about their health and care. For both healthcare scenarios, jurors voting for system B felt some degree of explainability would support patients with decreasing their risk of another stroke or increasing their chances of receiving a kidney. Similarly, for the non-healthcare scenarios, jurors emphasized the need for an explanation as a way for individuals to self-improve and enhance their future prospects.
2. *Learning opportunities for society*; learning opportunities for society included possibilities to improve the AI system itself through learning from the data and were primarily mentioned as an advantage of explainable AI in the non-healthcare context. Particularly for the criminal justice scenario, jurors discussed how an explanation would allow transparency of the decision-making process and potentially facilitate restorative justice.
3. *Ability to identify and resolve system biases*; overall, jurors discussed the risk of inherent bias in AI systems less extensively in the healthcare scenarios compared to the non-healthcare scenarios, with no concerns expressed for the stroke scenario. They did see this as a problem in the other 3 scenarios, mainly because the data used to develop AI systems in these contexts was more likely to be subjective or biased. For example, because people have to be well enough to receive a donor kidney, there would be limited data available on outcomes for those who are less well but may still benefit. Jurors felt that more explainable AI systems would best allow humans to address these biases and challenge decisions if they felt these to be unfair or based on biased data. At the same time, however, some jurors suggested that, for the non-healthcare scenarios, human decisions are often biased, and they saw AI systems as a potential solution to correct this.

#### Theme C: Trust in automated systems

1. *Fairness*; in the healthcare and recruitment scenarios, jurors considered more accurate systems fairer compared to more explainable systems because these contributed to equal access to stroke services or kidneys, or because AI systems would analyze data from all applicants equally, regardless of whether they had been successful or not. In the criminal justice scenario, jurors debated automated decision-making more passionately than for the other scenarios, preferring explainability when it came to fairness. They doubted if AI systems were suitable to fulfil such a complicated task without any human interference and flagged the long-term implications of incorrect decisions.
2. *Delivery of the decision*; this subtheme only emerged from juror discussions about the kidney transplantation and criminal justice scenarios. For the former, jurors were comfortable with less explainable decisions, but nevertheless regarded it an integral part of a doctor’s professional role to relay these decisions to patients and answer any questions. In the criminal justice context, jurors stressed that more explainable AI systems had the potential to facilitate a conversation about the decision.
3. *Accountability for decisions*; this subtheme only emerged for the healthcare scenarios, where jurors raised questions about who would be held responsible in case of incorrect automated decisions, and how accountability would be determined.

## DISCUSSION

### Main findings

We conducted two 5-day citizens’ juries in the UK with a representative sample of 36 participants to investigate how the public trades off accuracy and explainability of AI and whether this differs between healthcare and other scenarios. To our knowledge, our study is the first to engage citizens in public policy making on AI explainability in healthcare and compare this to other application domains.

We found that jurors favored accuracy over explainability in healthcare scenarios, but not in non-healthcare contexts where they either valued explainability equally to, or more than, accuracy. Their considerations for preferring accuracy regarded the potential positive impact of decisions on individuals and society and the potential to increase efficiency. Reasons for emphasizing explainability included opportunities for individuals and society to learn and improve, and an enhanced ability for humans to identify and address biases. Other considerations were related to trust in automated systems, including: fairness (with explanations considered crucial for fairness in criminal justice, but less so in other contexts); delivering the decision (primarily in criminal justice); and accountability (only in the healthcare context).

### Relation to other studies

In a survey among 170 physicians, Diprose et al^29^ found strong associations between AI explainability and physicians’ understanding of and trust in AI. They also reported that 88% of responding physicians preferred explainable over non-explainable AI, but without asking respondents to make the trade-off between explainability and accuracy. This may partly account for the stark difference with our findings. Furthermore, patients currently already rely on physicians to make decisions about their health and treatment, so trusting another entity in this context is not new to them. This is different for physicians, whose position as a decision maker changes when introducing decision support systems that do not provide explanations. Lastly, physicians may feel, and often are, accountable for their decisions and, therefore, might want to be able to explain AI system recommendations to patients. However, our results suggest that patients may value explainability differently, especially when weighed against a potential decrease in decision accuracy.

### Limitations

One limitation of our study was the difference in materials and witnesses between the healthcare and non-healthcare scenarios, which may have affected their comparability and how jurors made the trade-off for each of them. The healthcare scenarios included perspectives of witnesses who would be directly affected by decisions of the AI systems (ie, kidney patient waiting for a donor and a Stroke Association support worker), whereas the non-healthcare scenario witnesses were system users (ie, recruitment agency staff and criminal justice experts).

Both juries produced a report in their own words as part of the jury process, which are publicly available.^24^ Although the themes and deliberations in the jurors’ reports strongly aligned with the themes emerging from our post-jury analysis, we did not ask the jurors to confirm whether they thought our themes adequately reflected their perspectives (ie, member checking). This is another limitation of our study.

### Implications

The citizens in our juries made it clear that they cared more about accuracy than about explainability in healthcare scenarios compared to non-healthcare scenarios. Therefore, a categorical rule (eg, in legislation) that citizens are always entitled to explanations of AI decisions regardless of the circumstances may not be in keeping with citizens’ preferences and their best interests; particularly in cases of more advanced AI systems where it is difficult or impossible to offer good explanations without compromising accuracy. Our findings suggest that, instead of setting categorical rules around AI explainability, policy makers should consider making these domain-specific.

In the UK, our findings have informed new guidance,^30^ developed by the Information Commissioner’s Office (the UK’s independent body set up to uphold information rights) and the Alan Turing Institute (the UK’s national institute for data science and AI). This new guidance gives organizations practical advice on explaining the processes, services, and decisions delivered or assisted by AI to the individuals affected by them. It argues that to enhance the explainability of AI decisions, organizations should: be transparent, be accountable, consider the context in which they are operating, and reflect on the impact of the AI system on the individuals affected as well as the wider society.

In our study, we assumed a trade-off between accuracy and explainability of AI models. Generally speaking, more complex systems are capable of modeling complex decision functions more accurately but are less likely to be interpretable by people. However, not every decision problem in healthcare requires modeling of a complex function: sometimes relatively simple models can achieve similar accuracy levels.^31^ In such cases, there is no trade-off between accuracy and explainability. To account for a range of situations with trade-offs being more or less prominent, developers of healthcare decision support systems could consider incorporating a simpler model alongside a more complex one and present end users with the results of both. Finally, there may be ways to achieve the goal of explainability without end users necessarily having model logic completely explained or to reduce the need for explainability through alternative guarantees (eg, algorithmic fairness^32^) This is a field of ongoing investigation.^33^

### Unanswered questions

Despite strong consensus on the importance of explainability of AI decision-making, there exists significant variation in what is meant by this.^8^ Some authors assume it relates to data use, while others link it to access to source code, autonomy of the AI system, the extent to which it achieves causal understanding,^34^ or contestability of the system’s decision.^33^ In the context of our study, full explainability (system A) meant that it is always possible to trace the reasoning of a system from start to end. Partial explainability (system B) meant it is known which variables are important for decision-making but not how they are important. Future research may look into the trade-off with accuracy for other types of explainability and compare these with our findings.

AI explainability can be defined relative to different groups of stakeholders (eg, healthcare professionals, patients, regulators, policy makers)^35^ as well as to healthcare decisions (eg, stroke diagnosis) with and without immediate impact on life and death (eg, prognosis). The trade-off between accuracy and explainability is likely to be relevant to all these stakeholders and in all these decisions, but they might make the trade-off differently—particularly when presented with a variety of scenarios. Future studies should, therefore, investigate the views of these stakeholder groups on a broader range of scenarios.

## CONCLUSION

Citizens may value explainability of AI systems in healthcare less than in non-healthcare contexts and less than often assumed by healthcare professionals and researchers, especially when weighed against system accuracy. Our findings thus warrant active consultation of the public when developing policy on AI explainability in healthcare settings.

## FUNDING

This work was funded by the National Institute for Health Research Greater Manchester Patient Safety Translational Research Centre (NIHR GM PSTRC) and the Information Commissioner’s Office. The views expressed are those of the author(s) and not necessarily those of the NHS, the NIHR or the Department of Health and Social Care. NIHR had no role in study design, data collection, data analysis, data interpretation, or writing of the report. The corresponding author has full access to all of the data and the final responsibility to submit for publication.

## AUTHOR CONTRIBUTIONS

NP and MO conceived the idea for the study. MT, KB, SA, AH, and CW helped to develop the idea. KB and SA designed and facilitated both jury events. MO collected and analyzed quantitative data, while LR, SHS, and DP collected and analyzed qualitative data. SvdV helped to interpret the results. SvdV, LR, and NP drafted the manuscript with important intellectual input from all authors, who approved the final version of the manuscript for publication.

## DATA AVAILABILITY STATEMENT

The data underlying this article will be shared on reasonable request to the corresponding author.

## SUPPLEMENTARY MATERIAL

Supplementary material is available at *Journal of the American Medical Informatics Association* online.

## Supplementary Material

ocab127_Supplementary_Data
