# Supplementary material for: Trading off accuracy and explainability in AI decision-making: findings from 2 citizens’ juries
Source: J Am Med Inform Assoc. 2021 Aug 1;28(10):2128–38. doi: 10.1093/jamia/ocab127 (PMC8522832; doi:10.1093/jamia/ocab127)
Supplement: ocab127_Supplementary_Data [file ocab127_supplementary_data.pdf]

# Supplementary materials

## Supplement Part 1: Detailed 5-day jury program

Day 1

Jurors were given an introductory presentation on the citizens' jury method and guidelines for productive discussions and deliberation, followed by an exercise to practice effective group working on a policy problem. This was followed in the afternoon by a screening of the BBC documentary "The Joy of AI", and an explanation from an expert witness on balancing AI and explainability.

Day 2

This day started with an expert witness presentation on the law concerning data protection and AI, followed by a plea from a partial expert witness for maximising AI performance and a plea from another partial expert witness for transparent and explainable AI.

Days 3 and 4

The jurors discussed the four scenarios involving applications of AI (see Supplement 2), two of which were related to healthcare (diagnosis of stroke; risk assessment of kidney rejection after transplantation) and two of which were not (job recruitment; criminal justice). The relevant information was provided by the scenario witnesses. In each of the four scenarios, jurors were given descriptions of three types of automated decision-making systems with different levels of accuracy and explainability (system A, expert system; system B, random forest model; system C, deep neural network).

Following the plenary sessions in which scenarios and systems were explained, jurors were allocated at random into groups of six, working at three tables representing systems A, B and C, and rotating to each of these in turn to ensure they had an understanding of all three systems for each scenario. Whilst at each table, jurors independently wrote down advantages and disadvantages for each system and then agreed these collaboratively as a group. During group feedback, additional points were added by subsequent groups to flip charts, and eventually all jurors individually selected what they thought was the most important factor for each system. Finally, jurors electronically voted for their preferred system for each of the four scenarios. Free text boxes allowed up to three reasons to support their choice.

Day 5

Jurors voted on more general (non-scenario specific) questions about explainability of AI, including 'What matters most to you – accuracy or explainability?' and 'When/why are explanations important?' providing free text to substantiate their choice. The results of the Day 3/4 electronic voting and reasons for choices for each scenario formed input for a draft report which was presented back to the jurors.

## Supplement Part 2: Detailed scenarios as provided to the jurors

**HEALTHCARE SCENARIOS**

Stroke

There are more than 100,000 strokes in the UK each year – that is around one stroke every five minutes. About 11% of patients die immediately or within a few weeks as a result of the stroke, making stroke the fourth biggest killer in the UK. Almost two thirds of stroke survivors leave hospital with a disability.

*System A – Expert System*: 75% (non-expert level, e.g. A&E doctor) accuracy, full explanation
This system uses an algorithm that was developed with help from experienced neurologists and neuroradiologists, and aims to follow the same reasoning as they would do. In practice it does not reach the same level of accuracy as they would, but the algorithm is completely transparent in the way it reaches its conclusions: for each individual case it can provide specific rules that were applied to reach a conclusion. It has an overall accuracy rate of 75%, which is comparable to what most emergency medicine doctors would achieve. This means that in 25% of cases, someone might be classified as having a stroke while they were not or vice versa, or the type, location, and severity of the stroke might be misjudged.·

*System B – Random Forest system:* 85% (human expert level) accuracy, partial explanation
This system uses an algorithm that was established through machine learning from a large set of patient data, collected at English hospitals. This algorithm reaches (human) expert level performance, but it is not very transparent in the way it reaches its conclusions: it can only tell us which features, in general, are important and which are not. It has an overall accuracy rate of 85%. This means that in 15% of cases, someone might be classified as having a stroke while they were not or vice versa, or the type, location, and severity of the stroke might be misjudged.·

*System C – Deep Learning system:* 95% (beyond human level) accuracy, no explanation.
This system uses advanced AI derived from the same set of patient data as System B. However it has "taught itself" from the data which features were best able to distinguish strokes from non-strokes, and best able to distinguish different types of stroke, their location, and their severity. This algorithm is not transparent in the way it reaches conclusions: it is unable to provide any explanation that is understandable by humans. However it has an overall accuracy rate of 95%, which is better than human experts perform. This means that in 5% of cases, someone might be classified as having a stroke while they were not or vice versa, or the type, location, and severity of the stroke might be misjudged.

Kidney transplantation

It is hoped that with this system, a larger number of transplanted kidneys will survive longer. There are three automated decision systems to choose from – system A, system B, and system C.

*System A – Expert system*: 75% (below human expert level) accuracy, full explanation
This system uses an algorithm that was developed with help from experienced kidney doctors, and aims to follow the same reasoning as they would do. In practice it does not reach the same level of accuracy as they would, but the algorithm is completely transparent in the way it reaches its conclusions: for each individual case it can provide specific rules that were applied to reach a conclusion. It has an overall accuracy rate of 75%, which is a little lower than what is currently achieved in practice across the NHS (and lower than that achieved by the top specialists). This means that 25% of the time its predictions were incorrect (e.g. predicting that the kidney would last at least 5 years for the selected patient when in reality it didn't).·

*System B – Random Forest system:* 85% (human expert level) accuracy, partial explanation
This system uses an algorithm that was established through machine learning from a large set of patient data, collected at English hospitals. This algorithm achieves (human) expert level performance, but it is not very transparent in the way it reaches its conclusions: it can only tell us which features, in general, are important and which are not. It has an overall accuracy rate of 85%. This means that 15% of the time its predictions were incorrect (e.g. predicting that the kidney would last at least 5 years for the selected patient when in reality it didn't).

*System C – Deep Learning system:* 95% (beyond human level) accuracy, no explanation
This system uses advanced AI, derived from the same set of patient data as System B. However it has "taught itself" from the data which features were best able to distinguish successful matches from non-successful matches. This algorithm is not transparent in the way it reaches conclusions: it is unable to provide any explanation that is understandable by humans. However it has an overall accuracy rate of 95%, which is better than human experts perform. This means that 5% of the time its predictions were incorrect (e.g. predicting that the kidney would last at least 5 years for the selected patient when in reality it didn't.

**NON-HEALTHCARE SCENARIOS**

Recruitment

*System A – Expert system*: 75% (non-expert level, e.g. recruitment officer) accuracy, full explanation
This system uses an algorithm that was developed with help from experienced recruitment officers, and aims to follow the same reasoning as they would do. In practice it does not reach the same level of accuracy as they would, but the algorithm is completely transparent in the way it reaches its conclusions: for each individual case it can provide specific rules that were applied to reach a conclusion. When tested on existing data about recruitment, this system was shown to have an overall accuracy rate of 75%. This means that 25% of the time its predictions were incorrect (e.g. predicting that an applicant would be unlikely to become a high performing employee when in reality they did, or vice versa).The accuracy of this system is comparable to that of a typical recruitment officer.

*System B – Random Forest system*: 85% (human expert level) accuracy, partial explanation
This system uses an algorithm that was established through machine learning from a large set of recruitment data, collected by the organisation. This algorithm achieves (human) expert level performance, but it is not very transparent in the way it reaches its conclusions: it can only tell us which features, in general, are important and which are not. When tested on existing data about recruitment, this system was shown to have an overall accuracy rate of 85%. This means that 15% of the time its predictions were incorrect (e.g. predicting that an applicant would be unlikely to become a high-performing employee when in reality they did, or vice versa).The accuracy of this system is comparable to that of a very experienced recruitment officer.

*System C – Deep Learning system:* 95% (beyond human level) accuracy, no explanation
This system uses advanced AI, derived from the same set of data as System B. However it has "taught itself" from the data. This algorithm is not transparent in the way it reaches conclusions: it is unable to provide any explanation that is understandable by humans. However it has an overall accuracy rate of 95%, which is better than human experts perform. This means that 5% of the time its predictions were incorrect (e.g. predicting that an applicant would be unlikely to become a high performing employee when in reality they did, or vice versa).

Criminal justice

There are 3 automated decision systems for the Police to choose from:·

*System A – Expert system:* 75% (non-expert level, e.g. custody officer) accuracy, full explanation
This system uses an algorithm that was developed with help from very experienced Police Custody Officers, and aims to follow the same reasoning as they would do. In practice it does not reach the same level of accuracy as they would, but the algorithm is completely transparent in the way it reaches its conclusions: for each individual case it can provide specific rules that were applied to reach a conclusion. When tested on existing data about reoffending, this system was shown to have an overall accuracy rate of 75%. This means that 25% of the time its predictions were incorrect (e.g. predicting that an individual would commit a serious offence when in reality they didn't, or vice versa).The accuracy of this system is comparable to that of an average Police Custody Officer.·

*System B – Random Forest system:* 85% (human expert level) accuracy, partial explanation
This system uses an algorithm that was established through machine learning from a large set of criminal offence data, collected by the police and local agencies. This algorithm achieves (human) expert level performance, but it is not very transparent in the way it reaches its conclusions: it can only tell us which features, in general, are important and which are not. When tested on existing data about reoffending, this system was shown to have an overall accuracy rate of 85%. This means that 15% of the time its predictions were incorrect (e.g. predicting that an individual would commit a serious offence when in reality they didn't, or vice versa.)The accuracy of this system is comparable to that of a very experienced Police Custody Officer.·

*System C – Deep Learning system:* 95% (beyond human level) accuracy, no explanation
This system uses advanced AI, derived from the same set of data as System B. However it has "taught itself" from the data. This algorithm is not transparent in the way it reaches conclusions: it is unable to provide any explanation that is understandable by humans. However it has an overall accuracy rate of 95%, which is better than human experts perform. This means that 5% of the time its predictions were incorrect (e.g. predicting that an individual would commit a serious offence when in reality they didn't, or vice versa.)

## Supplement Part 3: Worked example of the qualitative analysis steps

The process of how the qualitative research team went from initial coding to theme generation is illustrated by the following quote from the stroke scenario *"Who is responsible if the diagnosis is wrong with fatal consequences*?". This excerpt was initially attributed to a theme called 'Accuracy/error', but subsequently re-coded to 'Accountability for decisions' following the first coding meeting. The concept of AI making decisions but not being liable in the same way as a person was pivotal within the third theme, labelled 'Trust'.  This theme was initially termed 'Human element', as we felt the AI systems were being conferred with the human traits expected of doctors in the medical scenarios. It was subsequently amended to 'Trust in automated systems' alongside the other subthemes 'Fairness' and 'Delivery of the decision'.

## Supplement Part 4: Questions and other points raised by jurors during the plenary sessions for each scenario; all were addressed prior to voting

| ***Scenario*** | ***Question*** | ***Topic*** |
|---|---|---|
| | *Healthcare scenarios* | |
| **Stroke** | Who would control the data going into deep learning? | Ownership |
| | Is the objective of AI in the NHS to improve patient care or decrease costs? | Reason |
| | NHS data has been hacked previously, could data be changed – what measures would be put in place? | Data security |
| | If we know data can be biased if that's found to be the case can bias be corrected? | Bias |
| | Whilst no explanation for patient, is there an opportunity for developers and people wanting to learn from diagnoses to inform public health or research? | Learning |
| | How does use of system C – deep learning sit with medical code of practice? | Human interaction |
| | How often can deep learning systems be updated, can they update themselves? | Learning |
| **Kidney transplantation** | Does a match mean two people get kidneys? | Resources |
| | When there is a match between three people all with the same attributes and for arguments sake same age – who gets the kidney? | Benefit |
| | Would doctors need information about how the match was made in order to determine medications required post-transplant? | Learning |
| | Can doctors correlate this (system outputs) with other factors? | Learning |
| | There are the same confidence levels between systems B&C – is that relevant- (Clarification that confidence isn't about accuracy) | Clarification |
| | Feels hard to measure if successful match or not – Drs can make mistake, so can patients, AI could be 100% perfect but people not make optimal decisions and AI be blamed | Accuracy |
| | Can't expect things to go back onto the individual – it required individual to change lifestyle and not blame computer | Benefit |
| | What if system C matches similar people, who decides? | Human interaction |
| | If NHS chose system C would more kidney transplants be performed? | Resources |
| | If younger patients are prioritised is that data included in the system? Blood pressure and type are objective, age seems more subjective so ethically different | Bias |
| | If a man has to stay within 2 hours of transplant hospital could the system tell him his % chance of a match? | Explanation |

| | | |
|---|---|---|
| | If a person is 55-60 years old will they have less chance of a kidney transplant than a 20 year old? | Bias |
| | Machine system is based on available data so there isn't any data from people not given a transplant previously. | Learning |
| | Could they start including that data if it became available? Could they start to collect it now? | Learning |
| | With AI systems already in use where a team previously made decisions and it includes consultant surgeons does it make the process more time efficient? Can they use that time to practice their skills and make transplantation better? | Resources |
| | If there's a really large pool of people all needing transplants but only a certain number of kidneys available could system C filter down to a list of low level risk but will the system then build in who is the most important? | Learning |
| | When ranking very good/adequate or poor match is age only factor or does it use these categories too? | Learning |
| *Non-healthcare scenarios* | | |
| **Recruitment** | Race was mentioned, could AI be trained to discriminate and be 'racist'? | Bias |
| | How do you measure success / failure - Not as easy as in the medical scenarios. | Accuracy |
| | Will the NHS or are they currently using AI to recruit? | Reason |
| | The use of AI in this context is entirely context dependent, certain organisations will want 100s of a certain type of employees whilst others will want to sit at a table and hand pick them. | Benefit |
| | Do we answer these questions as the recruiter or from the service user aspect? My views are very different depending on this. | Benefit |
| | We depend on feedback to improve, if we use system C deep learning and receive no feedback…but people are given no feedback wont it make society have less feedback and therefore less chance to improve? | Learning |
| **Criminal justice** | Say the scenario was about a first time offender they weren't on any database – if they went to court and were found guilty the court might rule for them to have rehab. If the person was sent straight to rehab this route would they not have a record and do they have any choice in this? | Bias; human interaction |
| | This person is innocent until proven guilty. I'm not happy sending person on rehab if not been found guilty of any offence. | Bias; learning |
| | If someone has a criminal record it is more likely they reoffend. By system sending person to trial they are then more likely to have a record. | Reason |
| | Could system C be used in offender profiling or to predict future behaviour? | Learning |

## Supplement Part 5: Electronic voting results per scenario per jury location

In the tables below, each row shows how jurors of a particular jury voted for a particular scenario. Individual jurors could vote only once per scenario. Values refer to the number of jurors (out of 18 in total) voting for that option.

*Q1: How important it is for an individual to receive an explanation of an automated decision?*

| | | ***Very important*** | ***Fairly important*** | ***Not very important*** | ***Not at all important*** | ***Don't know*** |
|---|---|---|---|---|---|---|
| *Healthcare scenarios* | | | | | | |
| **Stroke** | Coventry | 1 | 3 | 10 | 4 | 0 |
| | Manchester | 3 | 3 | 11 | 0 | 1 |
| **Kidney transplantation** | Coventry | 1 | 7 | 8 | 2 | 0 |
| | Manchester | 0 | 5 | 10 | 3 | 0 |
| *Non-healthcare scenarios* | | | | | | |
| **Recruitment** | Coventry | 4 | 9 | 5 | 0 | 0 |
| | Manchester | 4 | 7 | 6 | 1 | 0 |
| **Criminal justice** | Coventry | 10 | 3 | 5 | 0 | 0 |
| | Manchester | 7 | 6 | 3 | 2 | 0 |

*Q2: If system C was chosen [by the NHS], almost no explanation would be provided. How much does this matter?*

| | | ***Very much*** | ***Quite a lot*** | ***Not very much*** | ***Not at all*** | ***Don't know*** |
|---|---|---|---|---|---|---|
| *Healthcare scenarios* | | | | | | |
| **Stroke** | Coventry | 2 | 1 | 12 | 3 | 0 |
| | Manchester | 2 | 4 | 12 | 0 | 0 |
| **Kidney transplantation** | Coventry | 1 | 2 | 8 | 7 | 0 |
| | Manchester | 0 | 1 | 12 | 5 | 0 |
| *Non-healthcare scenarios* | | | | | | |
| **Recruitment** | Coventry | 7 | 7 | 4 | 0 | 0 |
| | Manchester | 3 | 5 | 9 | 1 | 0 |
| **Criminal justice** | Coventry | 11 | 1 | 5 | 1 | 0 |
| | Manchester | 6 | 8 | 2 | 2 | 0 |

*Q3: Which automated decision system[a)] do you think the NHS should choose/ should be chosen?*

| | | ***System A*** | ***System B*** | ***System C*** |
|---|---|---|---|---|
| *Healthcare scenarios* | | | | |
| **Stroke** | Coventry | 0 | 2 | 16 |
| | Manchester | 0 | 3 | 15 |
| **Kidney transplantation** | Coventry | 1 | 2 | 15 |
| | Manchester | 0 | 0 | 18 |
| *Non-healthcare scenarios* | | | | |
| **Recruitment** | Coventry | 2 | 14 | 2 |
| | Manchester | 5 | 6 | 7 |
| **Criminal justice** | Coventry | 8 | 7 | 3 |
| | Manchester | 7 | 6 | 5 |

a) System A, expert system (below human expert-level accuracy, full explanation); System B, random forest system (human expert-level accuracy, partial explanation); System C, deep learning system (beyond human expert-level accuracy, no explanation)

## Supplement Part 6: Statements captured during small group discussions on (dis)advantages of the system receiving the most votes for a particular scenario.

Statements are organised per (sub)theme from our qualitative analysis. The N provided for statements refers to the number of participants in that jury who selected that statement as being the most important; if none of the participants selected that statement as being the most important, no N is provided.

**HEALTHCARE SCENARIOS**Stroke (System C: beyond human expert-level accuracy, no explanation)

| ***Subthemes*** | ***Advantages*** | ***Disadvantages*** |
|---|---|---|
| | *ACCURACY* | |
| *Impact on individual* | • "Initially accuracy of the diagnosis is more important than the explanation" [Manchester, N=10]<br>• "Higher diagnosis success rate using same symptoms presented by the patient on initial consultation" [Coventry, N=8]<br>• "More accurate and has only 5% chance of misjudgement" [Manchester, N=7]<br>• "Accuracy would bring improvements to patient care and data" [Coventry, N=4]<br>• "Will save lives" [Manchester] | • "It treats disease as a solo issue to be fixed and ignores the benefit of holistic diagnosis" [Coventry, N=2] |
| *Impact on society* | • "It would be able to treat a higher number of patients and possibly free up more time for doctors/experts to treat other patients or do more research" [Coventry, N=3] | • "More patients will survive, have we got the infrastructure in place / funding to care for them?" [Coventry, N=2] |
| *Increased efficiency* | • "Speeds up access to treatments, care and support" [Coventry, N=4]<br>• "More efficient and therefore cost effective" [Coventry]<br>• "Less manpower is needed for an already overstretched NHS" [Manchester] | • "Reduces the number of trained and competent staff" [Manchester, N=2] |
| | *EXPLAINABILITY* | |
| *Learning for individuals* | • "People do not always relate to how a diagnosis is made, but what the diagnosis is, which can still be provided" [Manchester] | • "No explanation can be given as to how the decision was reached…" [Coventry, N=10]<br>• "5% diagnoses are inaccurate which could lead to deaths without explanations" [Manchester, N=3] |

| | | |
|---|---|---|
| | | • "The machines could become too intelligent and use their own language which consultants cannot understand or interpret" [Manchester, N=1] |
| *Learning for society* | • "Global solution through shared information / AI" [Coventry] | • "System C could overload and crash" [Manchester, N=4]<br>• "Can't interrogate the system so can't improve the result" [Manchester, N=2] |
| *Ability to identify/resolve bias* | | • "Experts could start to display bias in presuming it is correct" [Manchester, N=4]<br>• "Hacks could be untraceable due to not knowing how it reaches a decision and the high amount of data present" [Coventry, N=2]<br>• "What we deem accurate may still be based on bad or incomplete data sets restricted by GDPR or catchment of people analysed and information fed into the system" [Manchester] |
| | *TRUST* | |
| *Accountability* | | "Who is responsible if the diagnosis is wrong with fatal consequences?" [Coventry, N=2] |

<u>Kidney transplantation</u> (System C: beyond human expert-level accuracy, no explanation)

| **Subthemes** | **Advantages** | **Disadvantages** |
|---|---|---|
| | *ACCURACY* | |
| *Impact on individual* | • "95% accuracy rate and patients are less likely to ask how they are matched. When you win the lottery you just need to know you won, not which order the balls come up" [Coventry, N=11]<br>• "All you need to know is if it's a match or not – no explanation is needed" [Manchester, N=1] | • "Not preferable because of no explanation at all" [Manchester, N=2] |
| *Impact on society* | • "The greater degree of accuracy reduces the number of failures [rejections] feeding back into the system – ultimately reducing waiting lists" [Manchester, N=11]<br>• "95% offers a low risk outcome …benefits the NHS and patients" [Coventry, N=3]<br>• "Provides the greatest scope for accuracy" [Manchester, N=3] | • "We only know the outcome for the people who have been selected" [Manchester, N=8]<br>• "It could widen health inequalities" [Coventry, N=4] |

| | | |
|---|---|---|
| *Increased efficiency* | • "Accuracy / speed of decision is preferable because of less likelihood of rejection" Coventry (4G)<br>• "Preferable because it maximises a valuable resource [the kidney]" Manchester (3G) | • "Is there enough kidney donors to warrant using an AI system (cost) 8000 Patients: donors ratio?" [Coventry] |
| | *EXPLAINABILITY* | |
| *Learning for individuals* | | • "No transparency to show the criteria used (BMI, Blood Pressure, lifestyle) factored into the decision" [Coventry, N=10] |
| *Ability to identify/resolve bias* | | • "Any automated bias is not transparent and can't be discerned" [Manchester, N=6]<br>• "We do not yet know about the effects of bias in data / database" [Coventry, N=4]<br>• "The software could be 'gamed' so that certain people get transplants and it would be hard to identify" [Coventry] |
| | *TRUST* | |
| *Delivering the decision* | | • "System C is not preferable because of its inability to provide empathy and sympathy to patients" [Coventry]<br>• "It takes away human emotion from the decision" [Manchester] |

**NON-HEALTHCARE SCENARIOS**

<u>Recruitment</u> (System B: human expert-level accuracy, partial explanation)

| **Subthemes** | **Advantages** | **Disadvantages** |
|---|---|---|
| | *ACCURACY* | |
| *Impact on individual* | • "It has key points/ features for detection. Applicant receives some feedback" [Coventry, N=15]<br>• "It offers a high level of accuracy and gives some explanation / feedback" [Manchester, N=9] | • "Less transparent feedback – e.g. could tell you that you didn't meet essential criteria but not tell you which part you did not meet to improve future chances of success" [Coventry , N=12]<br>• "Less accurate than [system] C" [Manchester, N=2] |
| *Impact on society* | • "The algorithm achieves human expert level" [Manchester, N=6]<br>• "Human level performance 85% high possibility of hiring right candidate" [Coventry, N=1]<br>• "Accuracy is comparable to that of a very experienced recruiter" | • "Less trust in the company" [Coventry] |

| | | |
|---|---|---|
| | [Coventry] | |
| *Increased efficiency* | | • "Over-reliance in the AI system means fewer high level HR managers are involved in the recruitment process" [Manchester, N=1] |
| | *EXPLAINABILITY* | |
| *Learning for individuals* | | • "Only offers partial feedback" [Manchester, N=1]<br>• "Limited feedback may not be used" [Coventry] |
| *Learning for society* | | • "Quality of the data is too variable and unreliable" [Coventry, N=3] |
| *Ability to identify/resolve bias* | • "It is on par with human performance but reduces the possibility of human bias" [Manchester, N=5]<br>• "Has the ability to explicitly pick up biases based on rules / tree forests" [Coventry , N=2] | • "Historical data use introduces a level of automated bias" [Manchester, N=13] |
| | *TRUST* | |
| *Fairness* | • "It is 'democratic' in its analysis of past applicants, both successful and unsuccessful, without developing unknowable deep learning iterations" [Manchester, N=2] | |

<u>Criminal justice</u> (System A: below human expert-level accuracy, full explanation)

| **Subtheme** | **Advantages** | **Disadvantages** |
|---|---|---|
| | *ACCURACY* | |
| *Impact on individual* | | • "It is the least accurate [system] 25% incorrect predictions is costly for those who go to rehab or wrongful prosecution" [Coventry, N=6] |
| *Impact on society* | | • "Less accurate than [system] B or C [Manchester, N=4]<br>• "Less accurate than a very experienced police custody officer" [Manchester] |
| *Increased efficiency* | | • "It would require a lot of work from software engineers and policemen" [Coventry, N=3] |
| | *EXPLAINABILITY* | |
| *Learning for individuals* | • "The person is able to understand why / why not they have been offered rehab" [Coventry] | |
| *Learning for society* | • "Because the rules can be changed and so the system is flexible" [Coventry, N=2] | • "Has no ability to learn from data" [Manchester, N=4]<br>• "Data might be corrupt and thus a |

| | | |
|---|---|---|
| | • "Identification will identify other courses of action" [Manchester] | higher risk to the public!" [Coventry, N=3]<br>• "There is little to no machine learning from the data, therefore the system cannot evolve" [Coventry] |
| *Ability to identify/resolve bias* | • "It is fully transparent and bias can be clearly identified allowing human intervention / appeal of decision" [Coventry, N=15]<br>• "It carries no historical bias, which systems B&C will inevitably inherit from the collected data" [Manchester, N=8]<br>• "It is based on input by police experts instead of historical data – that is why it is more current" [Manchester] | • "Poor data based on previous mis-handling of individual cases would bias the system" [Coventry, N=6]<br>• "Some police officers could be biased towards individuals" [Manchester, N=3] |
| | *TRUST* | |
| *Fairness* | | • "Too much power is given to the staff who design or implement the rules in the system" [Manchester, N=7]<br>• "It is impossible to predict individual behaviour" [Coventry] |
| *Delivering the decision* | • "There is more interaction with officers. Feelings and remorse will be taken into account and a full explanation will be given for the end result" [Manchester, N=10]<br>• "It offers better dialogue between the authority and the offender with clearly defined parameters" [Coventry, N=1]<br>• "Lower accuracy but gives the best human touch to the process" [Coventry] | |
| *Accountability* | • "Transparent system is held to account" [Manchester] | |
